# *ATM* gene polymorphisms are associated with poor prognosis of non-small cell lung cancer receiving radiation therapy

**DOI:** 10.18632/aging.103094

**Published:** 2020-04-24

**Authors:** Junjun Mou, Tao Hu, Zhiwu Wang, Wei Chen, Yang Wang, Wei Zhang

**Affiliations:** 1 Department of Radiotherapy, Yantai Yuhuangding Hospital, The Affiliated Hospital of Qingdao University, Yantai 264000, Shandong, China; 2 Department of Thoracic Surgery, Yantai Yuhuangding Hospital, The Affiliated Hospital of Qingdao University, Yantai 264000, Shandong, China; 3 Department of Chemoradiotherapy, Tangshan People’s Hospital, Tangshan 063000, China; 4 Training Department, Yantai Yuhuangding Hospital, The Affiliated Hospital of Qingdao University, Yantai 264000, Shandong, China

**Keywords:** ataxia telangiectasia mutated gene, non-small cell lung cancer, polymorphism, prognosis, radiation therapy

## Abstract

We investigated the prognostic significance of *ataxia telangiectasia mutated* (*ATM*) single nucleotide polymorphisms (SNPs) in 720 Han Chinese non-small cell lung cancer (NSCLC) patients who underwent radiation or chemoradiation therapy. Kaplan-Meier survival curves showed that overall survival (OS) and disease-free survival (DFS) rates were significantly associated with two *ATM* SNPs, rs664143 and rs189037. Patients with the rs664143 GA or AA genotype had poorer DFS (hazard ratio (HR) = 1.40, 95% confidence interval (CI) = 1.05–1.86, *P* = 0.021) and OS (HR = 1.28, 95%CI = 1.12–1.78, *P* = 0.040) than those with the rs664143 GG phenotype. Patients with the rs189037 AG/GG genotypes had poorer prognoses than those with the rs189037 AA genotype (AG/GG *vs.* AA: DFS, HR = 1.44, 95%CI = 1.06–1.95, *P*=0.019; OS, HR = 1.16, 95%CI = 1.16–1.17–2.21, *P*=0.004). These results were confirmed by subgroup analysis based on clinical factors such as smoking, histology, tumor stage, treatment, and radiation dose, all of which were significantly associated with DFS and OS rates in NSCLC patients. These findings show that *ATM* rs664143 and rs189037 variants determine prognosis in NSCLC patients that have undergone radiation or chemoradiation therapies.

## INTRODUCTION

Lung cancer is one of the leading causes of cancer mortality worldwide, with nearly 2.1 million new lung cancer cases and 1.8 million lung cancer deaths reported in 2018 [1]. Lung cancer incidence in the United States has improved significantly in men, but remains high in the women [2]. In China, the incidence of lung cancer has increased by 26.9% annually, and the numbers of lung cancer patients have doubled every 10 to 15 years in the last 50 years [3]. Moreover, in the last three decades, the mortality rate of lung cancer has increased by 465%, and replaced liver cancer as the most common malignant tumor in China [4]. The two main categories of lung cancer are small cell lung cancer (SCLC) and non-small cell lung cancer (NSCLC); NSCLC accounts for nearly 87% of all lung cancer cases, about 75% of which are diagnosed in the advanced stages with poor prognosis [5]. The 5-year survival rate of lung cancer patients is low and ranges from 5% to 31% [6, 7]. Radiotherapy is one of the main treatment modalities, especially for NSCLC patients that are not amenable for surgery [8–10]. The prognostic indicators in NSCLC patients include TNM stage [11], serum levels of tumor markers [12], tumor size [13], blood glucose levels, and treatment modalities [14, 15].

*Ataxia telangiectasia mutated* (*ATM*) gene is located on chromosome 11q 22.3 and encodes for the ATM protein that plays a significant role in DNA repair and cell cycle regulation [16, 17]. Previous case-control studies in Caucasian and Han Chinese populations have shown that several *ATM* gene polymorphisms are associated with increased risk of lung cancer [18–20]. However, their prognostic significance has not been studied in detail. Therefore, in this study, we analyzed the prognostic significance of four *ATM* SNPs in 720 Han Chinese NSCLC patients that received radiation or chemo-radiation therapy.

## RESULTS

### General characteristics of the study population

We enrolled 720 NSCLC patients, including 335 males (46.5%) and 385 females (53.5%). We obtained their clinical and follow-up data to evaluate DFS and OS outcomes. Among these 720 patients, 497 (69.0%) were above 60 years, and 223 (31.0%) were ≤ 60 years of age. Furthermore, these patients included 370 (51.4%) smokers, 158 (21.9%) with squamous cell carcinoma, 358 (49.7%) with adenocarcinomas, and 204 (28.3%) with other cancer types. Moreover, 333 patients were diagnosed with stage IIIA and 387 with stage IIIB NSCLC. The cohort included 380 patients that received radiation therapy alone, whereas, the remaining 340 patients received chemoradiation therapy. While 357 patients received intensity-modulated radiation therapy (IMRT), 363 patients received three-dimensional conformal radiotherapy (3D-CRT) or another kind of radiotherapy. The median radiation dose received by this cohort was 65 Gy (419 patients received < 65 Gy and 419 received ≥ 65 Gy). The four *ATM* SNPs analyzed in this study were rs664143 (GG, GA and AA genotypes at nucleotide positions 148, 381 and 191, respectively), rs664677 (TT, TC, and CC genotypes at nucleotide positions 265, 282, and 173, respectively), rs189037 (AA, AG, and GG genotypes at nucleotide positions 172, 283, and 165, respectively), and rs373759 (TT, TC, and CC genotypes at nucleotide positions 227, 294 and 199, respectively). The baseline clinical characteristics of the study population are listed in Table 1.

**Table 1:** Relationship between clinical parameters and disease-free survival and overall survival in patients with NSCLC.

**Parameters**	**Category**	**Disease-free survival**					**Overall survival**				
		**MST**	**Event/Total**	***P^a^***	**HR (95%CI)**	***P^b^***	**MST**	**Event/Total**	***P^a^***	**HR (95%CI)**	***P^b^***
Age	≤60	22.5	116/223	0.851	1.00		42.8	84/223	0.445	1.00	
	>60	25.2	267/497		0.98 (0.79-1.22)	0.851	38.7	209/497		1.10 (0.86-1.42)	0.445
Gender	Female	28.9	224/385	0.003	1.00		44.5	169/385		1.00	
	Male	24.1	159/335		1.36 (1.11-1.67)	0.003	30.3	124/335	0.118	1.20 (0.95-1.52)	0.119
Smoking	No	-	136/350		1.00		57.9	110/350		1.00	
	Yes	21.0	247/370	<0.001	2.09 (1.70-2.58)	<0.001	34.9	183/370	<0.001	1.69 (1.33-2.15)	<0.001
Histology	SCC	24.5	92/158	0.130	1.00		52.9	57/158	0.058	1.00	
	ADC	23.1	191/358		1.08 (0.84-1.38)	0.567	36.4	158/358		1.40 (0.98-1.90)	0.068
	Other	28.9	100/204		0.84 (0.63-1.11)	0.224	31.7	78/204		1.02 (0.72-1.44)	0.910
Stage	IIIA	43.1	142/333	<0.001	1.00		52.9	119/333	0.005	1.00	
	IIIB	22.7	241/387		1.67 (1.36-2.05)	<0.001	36.7	174/387		1.39 (1.12-1.76)	0.006
Treatment	CRT	40.4	149/340	<0.001	1.00		52.7	122/340	0.013	1.00	
	RT	22.0	234/380		1.63 (1.33-2.01)	<0.001	37.8	171/380		1.34 (1.16-1.69)	0.014
Radiation technique	IMRT	28.9	170/357	0.006	1.00		42.7	136/357	0.378	1.00	
	3D-CRT and other	24.7	213/363		1.33 (0.99-1.62)	0.056	41.1	157/363		1.11 (0.88-1.40)	0.379
Does	≥65Gy	29.6	243/419	0.001	1.00		42.6	117/301	0.136	1.00	
	<65Gy	23.1	140/301		1.42 (1.15-1.75)	0.001	38.2	176/419		1.29 (1.25-1.51)	0.013

*P^a^*, Log-rank P; *P^b^*, univariate cox regression; MST, median survival time; RT, radiation therapy; CRT, chemoradiation therapy; SCC, squamous cell carcinoma.

ADC, adenocarcinoma; IMRT, Intensity Modulated Radiation Therapy; 3D-CRT, three-dimensional conformal radiotherapy.

### Clinical characteristics and prognosis of NSCLC patients

The median follow-up time was 36.4 months (range: 2.9–94.6 months) and 293 patients died before the last follow-up. The median DFS was 24.5 months, and 383 patients showed disease progression. Kaplan–Meier survival curve analysis showed that gender (*P* = 0.003), smoking (*P* < 0.001), TNM stage (*P* < 0.001), treatment modality (*P* < 0.001), and radiation dose (P = 0.001) were significantly associated with DFS and OS. Patients with stage IIIB tumors showed significantly shorter DFS (HR = 1.67, 95%CI = 1.36–2.05, *P* < 0.001) and OS (HR = 1.39, 95%CI = 1.12–1.76, *P* = 0.006) than stage IIIA lung cancer patients. The survival time was significantly longer for patients treated with chemoradiation therapy than those treated with radiation therapy alone (40.4 *vs.* 22.0 months, *P* < 0.001). Moreover, patients treated with radiation therapy alone had worse survival outcomes than those treated with combination of chemo and radiation therapy (HR = 1.34, 95%CI = 1.16–1.69, *P* = 0.014). The OS rates for smoking NSCLC patients was significantly shorter than the non-smoking NSCLC patients (HR = 1.69, 95%CI = 1.69-1.33–2.15, *P* < 0.001). Patients that received a radiation dose < 65 Gy showed worse DFS rates (HR = 1.42, 95%CI = 1.15–1.75, *P* = 0.001) and OS (HR = 1.29, 95%CI = 1.25–1.51, *P* = 0.013) than NSCLC patients that received a radiation dose ≥ 65 Gy.

### *ATM* SNPs and survival outcomes of NSCLC patients

Univariate analyses showed that two *ATM* SNPs, rs664143 and rs189037, as shown in Supplementary Figure 1, were significantly associated with DFS and OS, but, the other two *ATM* SNPs, rs664677 and rs373759, did not show any significant correlation with survival outcomes (Table 2).

**Table 2:** Associations of ATM gene with DFS and OS in patients with NSCLC.

**SNP**	**Disease-free survival**					**Overall survival**				
	**Event/No.**	**MST**	***P^a^***	**Adjusted HR (95%CI)**	***P^b^***	**Event/No.**	**MST**	***P^a^***	**Adjusted HR (95%CI)**	***P^b^***
rs664143										
GG	58/148	33.4	0.008	1.00		43/148	44.0	0.029	1.00	
GA	219/381	24.3		1.41(1.05-1.89)	0.023	166/381	37.8		1.22(1.17-1.72)	0.014
AA	106/191	22.7		1.38(1.01-1.91)	0.034	84/191	33.6		1.40(1.23-2.04)	0.024
Trend										
GG	58/148	33.4	0.002	1.00		43/148	44.0	0.023	1.00	
GA+AA	325/571	23.8		1.40(1.05-1.86)	0.021	250/572	35.9		1.28(1.12-1.78)	0.040
GG+GA	277/528	26.0	0.248	1.00		209/529	36.1	0.049	1.00	
AA	106/191	22.7		1.06(0.85-1.33)	0.596	84/191	33.6		1.20(0.93-1.55)	0.164
rs664677										
TT	142/265	25.5	0.929	1.00		115/265	42.7	0.670	1.00	
TC	152/282	24.1		1.11(0.88-1.40)	0.379	116/282	38.7		1.13(0.87-1.47)	0.348
CC	89/173	28.0		1.02(0.78-1.33)	0.874	62/173	32.7		0.97(0.71-1.32)	0.852
Trend										
TT	160/259	25.5	0.928	1.00		115/265	42.7	0.888	1.00	
TC+CC	241/455	24.5		0.97(0.77-1.22)	0.777	178/455	35.7		1.07(0.85-1.36)	0.575
TT+TC	294/547	28.0	0.766	1.00		231/547	33.4	0.465	1.00	
CC	48/79	24.5		0.88(0.67-1.15)	0.344	62/173	32.7		0.91(0.69-1.21)	0.531
rs189037										
AA	94/172	31.0	0.007	1.00		56/172	43.5	<0.001	1.00	
AG	221/383	23.8		1.48(1.18-2.03)	0.014	192/383	34.9		1.76(1.21-2.45)	0.001
GG	68/165	24.5		1.32(1.17-1.91)	0.012	45/165	33.5		1.23(1.13-1.83)	0.029
										
Trend										
AA	94/172	31.0	0.004	1.000		53/172	43.5	0.001	1.00	
AG+GG	289/548	24.1		1.44(1.06-1.95)	0.019	237/548	36.0		1.16(1.17-2.21)	0.004
AA+AG	315/555	24.7	0.523	1.00		248/555	37.8	0.145	1.00	
GG	68/165	24.5		0.98(0.75-1.29)	0.906	45/165	33.5		0.80(0.60-1.08)	0.141
rs373759										
TT	122/227	25.2	0.508	1.00		103/227	35.6	0.103	1.00	
TC	157/294	23.1		1.14(0.90-1.45)	0.286	117/294	34.9		1.08(0.82-1.41)	0.590
CC	104/199	29.0		0.99(0.76-1.30)	0.968	73/199	44.9		0.81(0.60-1.10)	0.814
Trend										
TT	122/227	25.2	0.908	1.00		103/227	35.6	0.489	1.00	
TC+CC	261/493	24.5		1.07(0.87-1.34)	0.496	190/493	41.8		0.96(0.75-1.22)	0.739
TT+TC	279/521	29.0	0.324	1.00		220/521	35.4	0.035	1.00	
CC	104/199	24.7		0.92(0.73-1.17)	0.505	73/199	44.9		0.78(0.60-1.02)	0.074
Number of risk allele*								
0	14/35	33.6	<0.001	1.00		7/35	-	0.002	1.00	
1	85/155	24.5		1.37(0.78-2.41)	0.279	58/155	52.9		1.59(0.73-3.49)	0.244
2	164/310	26.7		1.30(0.75-2.42)	0.138	137/310	36.9		1.82(0.85-3.89)	0.122
3	106/180	-		1.53(1.13-2.66)	0.004	81/180	35.5		2.01(1.13-4.35)	0.017
4	14/40	19.7		2.91(1.43-3.93)	<0.001	10/40	28.6		1.42(1.22-2.95)	<0.001

*P^a^*, Log-rank P; *P^b^*, cox regression for adjusting age, gender, smoking, histology, stage, treatment, radiation technique, does; MST, median survival time.

Trend: the prognosis showed an increased or decreased changed with the number of risk allele.

*Number of risk allele: For rs664143(A) and rs189037(G): 0(GG+AA), 1(GG+AG, GA+AA), 3(AA+AG, GA+GG), 4(AA+GG).

**Figure 1:**
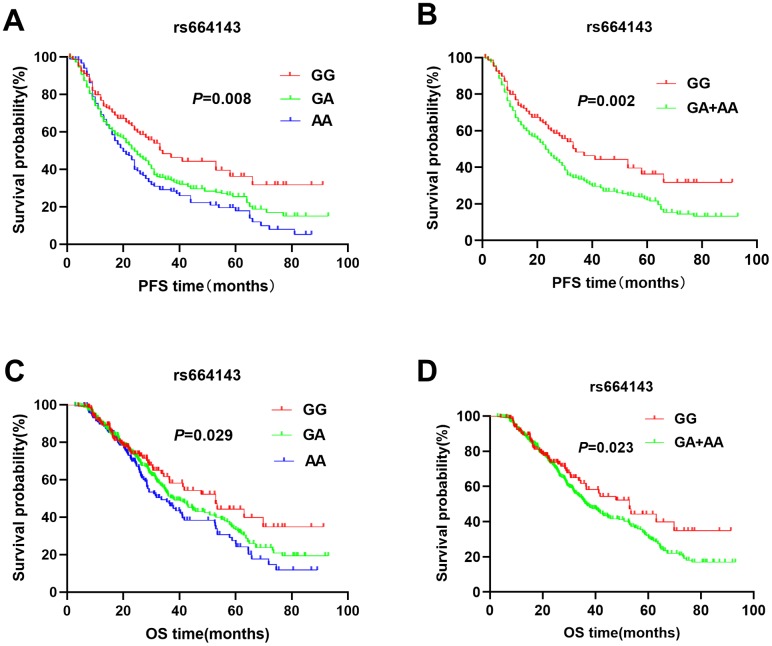
Kaplan-Meier survival curve analysis shows disease-free survival (**A**) GG vs. GA vs. AA, (**B**) GA/AA vs. GG); and overall survival (**C**) GG vs. GA vs. AA, (**D**) GA/AA vs. GG, of NSCLC patients with ATM rs664143 genotypes that are treated with radiation or chemoradiation therapies.

**Figure 2:**
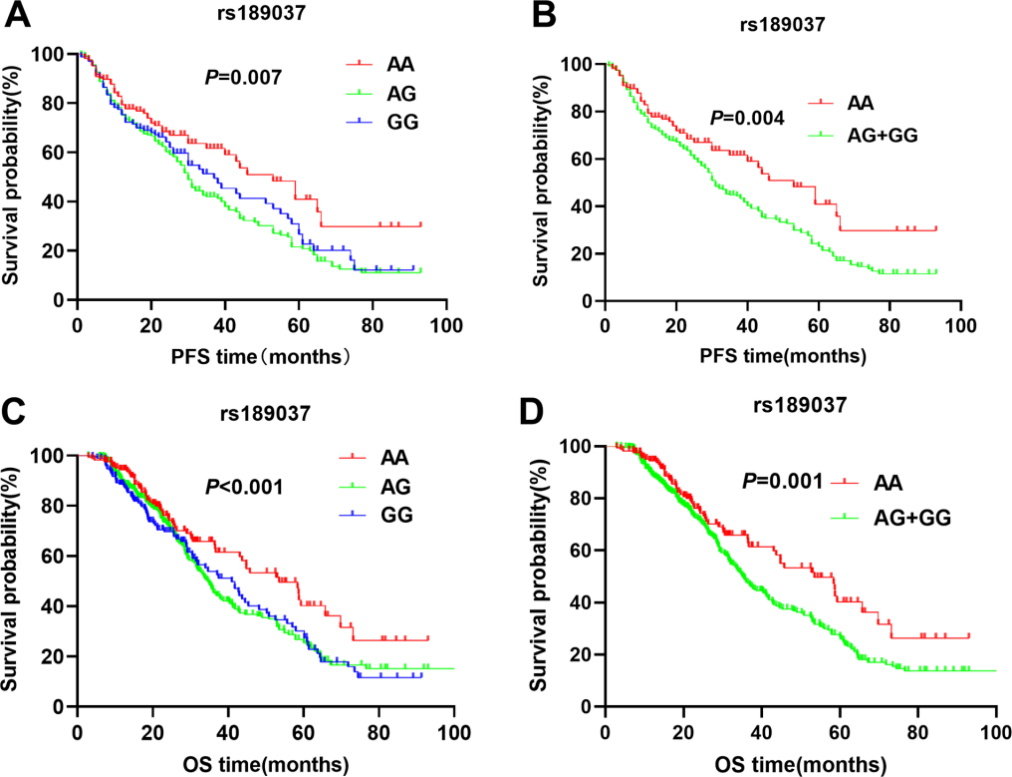
Kaplan-Meier survival curve analysis shows disease-free survival (**A**) AA vs. AG vs. GG, (**B**) AG/GG vs. AA; and overall survival (**C**) AA vs. AG vs. GG, (**D**) AG/GG vs. AA, of NSCLC patients with ATM rs189037 genotypes that are treated with radiation or chemoradiation therapies.

The patients with *ATM* rs664143 AA and GA genotypes showed significantly shorter DFS (median survival time or MST (month): 22.7 vs. 24.3 *vs.* 33.4; *P* = 0.008; Figure 1A) and OS (MST: 33.6 vs. 37.8 *vs.* 52.9 months; *P* = 0.029; Figure 1C) than patients with the rs664143 GG genotype. After adjusting for clinical parameters, Cox regression analysis showed that patients with *ATM* rs664143 AA or GA genotypes showed worse survival outcomes than those with the rs664143 GG phenotype (AA vs. GG, HR: 1.38, 95%CI = 1.01-1.19, *P* = 0.034; GA vs. GG, 1.41, 95%CI = 1.05–1.89, *P* = 0.023). The rs664143 G allele showed dominant effect than the rs664143 A allele (GA/AA *vs.* GG: *P* = 0.002 for DFS, Figure 1B; *P* = 0.023 for OS, Figure 1D). According to multivariate Cox regression analysis, patients with rs664143 GA and rs664143 AA genotypes showed worse DFS (HR = 1.40, 95%CI = 1.05–1.86, *P* = 0.021) and OS (HR = 1.28, 95%CI = 1.12–1.78, *P* = 0.040) than those with the rs664143 GG phenotype. Patients with the *ATM* rs189037 GG and rs189037 AG genotypes showed significantly shorter DFS (MST: 24.5 vs. 23.8 vs. 31.0 months, *P* = 0.007, Figure 2A) and OS (MST: 33.5 vs. 34.9 vs. 43.5, *P* < 0.001, Figure 2C) than those with the rs189037 AA genotype. After adjusting for clinical parameters, Cox regression analysis showed that patients with rs189037 GG or AG genotypes had worse DFS (GG *vs.* AA: HR = 1.32, 95%CI = 1.17–1.91, *P* = 0.012; AG *vs.* AA: HR = 1.48, 95%CI = 1.18–2.03, *P* = 0.014; Table 2) and OS (GG *vs.* AA: HR = 1.23, 95%CI = 1.13–1.83, P = 0.029; AG *vs.* AA: HR = 1.76, 95%CI = 1.21–2.45, *P* = 0.001) than those with rs189037 AA. The rs189037 G allele exhibited dominant effect than the rs189037 A allele (DFS: *P* = 0.004, Figure 2B; OS: *P* = 0.001, Figure 2D). Multivariate Cox regression analysis showed worse survival outcomes for patients with the rs189037 AG and GG genotypes than those with the rs189037 AA genotype (AG/GG *vs.* AA: DFS, HR = 1.44, 95%CI = 1.06–1.95, P = 0.019; OS, HR = 1.16, 95%CI = 1.16–1.17–2.21, *P* = 0.004; Table 2).

We also compared the association between the number of risk alleles (rs664143 and 189037) and survival outcomes, DFS and OS. The results showed that patients with the risk alleles were associated with worse DFS and OS compared to those without a risk allele (Table 2). As shown in Table 3, three SNPs (rs664143, rs664677, and rs373759) showed a prior false-positive probability of 0.1.

**Table 3:** False-positive reports probability values for associations between gene and survival outcomes.

**SNP**	**Disease-free survival**						**Overall survival**					
	**HR (95%CI)**	***P^b^***	**Statistical power**	**Prior probability**			**HR (95%CI)**	***P^b^***	**Statistical power**	**Prior probability**		
				**0.2**	**0.1**	**0.01**				**0.2**	**0.1**	**0.01**
rs664143												
GA vs. GG	1.41(1.05-1.89)	0.023	0.741	0.162	0.305	0.816	1.22(1.17-1.72)	0.014	0.634	0.106	0.301	0.764
AA vs. GG	1.38(1.01-1.91)	0.034	0.720	0.221	0.396	0.875	1.40(1.23-2.04)	0.024	0.821	0.035	**0.074**	0.612
GA/AA vs.GG	1.40(1.05-1.86)	0.021	0.712	0.111	0.198	0.801	1.28(1.12-1.78)	0.040	0.903	0.161	0.254	0.821
rs664677												
TCvs.TT	1.11(0.88-1.40)	0.379	0.701	0.221	0.297	0.897	1.13(0.87-1.47)	0.348	0.706	0.085	0.234	0.681
CC vs.TT	1.02(0.78-1.33)	0.874	0.623	0.047	0.110	0.654	0.97(0.71-1.32)	0.852	0.764	0.002	**0.097**	0.101
TC/CCvs.TT	0.97(0.77-1.22)	0.777	0.713	0.051	**0.055**	0.635	1.07(0.85-1.36)	0.575	0.934	0.001	0.216	0.024
rs189037												
GA vs. AA	1.48(1.08-2.03)	0.014	0.769	0.135	0.291	0.841	1.76(1.21-2.45)	0.001	0.638	0.006	0.204	0.883
GG vs. AA	1.32(1.17-1.91)	0.012	0.812	0.062	0.154	0.734	1.23(1.13-1.83)	0.029	0.694	0.012	0.114	0.154
GG/GA vs. AA	1.44(1.06-1.95)	0.019	0.934	0.216	0.221	0.886	1.16(1.17-2.21)	0.004	0.824	0.031	0.310	0.135
rs373759												
TC vs. TT	1.14(0.90-1.45)	0.286	0.685	0.253	0.442	0.885	1.08(0.82-1.41)	0.590	0.768	0.015	**0.014**	0.621
CC vs. TT	0.99(0.76-1.30)	0.968	0.821	0.131	0.241	0.769	0.81(0.60-1.10)	0.814	0.836	0.026	**0.004**	0.412
TC/CC vs. TT	1.07(0.87-1.34)	0.496	0.932	0.468	0.468	0.908	0.96(0.75-1.22)	0.739	0.931	0.018	**0.031**	0.232

### Subgroup analysis of *ATM* SNPs and NSCLC prognosis

We performed subgroup analysis with clinical factors such as smoking, histology, tumor stage, treatment, and radiation dose that are significantly associated with DFS and OS. The subgroup analysis showed that rs664143 and rs189037 were significantly associated with DFS and OS in both IIIA and IIIB stages (Table 4 and Table 5). NSCLC patients with *ATM* rs664143 GA and AA genotypes showed worse DFS (stage IIIA: adjusted HR = 1.34, 95%CI = 1.18–2.05, P = 0.022; stage IIIB: adjusted HR = 1.52, 95%CI = 1.04–2.22, *P* = 0.030) and OS (stage IIIA: adjusted HR = 1.29, 95%CI = 1.18–2.09, *P* = 0.028; stage IIIB: adjusted HR = 1.51, 95%CI = 1.17–2.37, *P* = 0.021) than patients with the rs664143 GG genotype. Stage IIIA and IIIB NSCLC patients with rs189037 AG/GG genotypes showed significantly shorter median survival times than those with the rs189037 AA genotype (Stage IIIA: 39.0 vs. 58.6 months; stage IIIB: 27.1 vs. 43.7 months; Table 4). Cox regression analysis after adjustment showed shorter DFS and OS for the rs189037 AG/GG genotypes in than those with the rs189037 AA genotype in stage IIIA NSCLC (DFS: adjusted HR, 1.43; 95% CI = 1.31–2.22, *P* = 0.037; OS: adjusted HR, 1.62;(95%CI = 1.02–2.57, *P* = 0.042; Table 5) and stage IIIB NSCLC (DFS: adjusted HR,1.55; 95% CI = 1.05–2.38, *P* = 0.029; OS: adjusted HR, 1.69; 95% CI = 1.09–2.65, *P* = 0.020; Table 5). Moreover, patients with rs664143 and rs189037 SNPs that were smokers and received radiation therapy (RT) with a radiation dose > 65 Gy showed worse survival outcomes than patients that were non-smokers and received chemo-radiation therapy (CRT) with a radiation dose ≤65 Gy, respectively (Tables 4, 5). Furthermore, based on histology, the rs664143 and rs189037 gene polymorphisms were associated with DFS and OS among the patients with adenocarcinomas (ADC) as shown in Tables 4, 5.

**Table 4:** Subgroup multivariate analysis of clinical parameters for rs664143 and survival outcomes.

**Parameters**	**Subgroup**	**Genotype**	**Disease-free survival**					**Overall survival**				
			**Event/No.**	**MST (month)**	***P^a^***	HR* (95%CI)	***P^b^***	**Event/No.**	**MST (month)**	***P^a^***	HR* (95%CI)	***P^b^***
rs664143												
Smoking	Yes	GG	34/62	24.1		1.00		27/62	36.4		1.00	
		GA	145/207	21.0	0.204	1.46(0.99-2.15)	0.057	105/207	35.6	0.838	0.98(0.63-1.51)	0.930
		AA	68/101	17.0	0.013	1.65(1.32-2.07)	0.026	51/101	21.2	0.023	1.10(1.09-1.77)	0.037
		GA+AA	213/308	19.5	0.033	1.42(1.06-2.08)	0.047	156/308	29.4	0.014	1.31(1.27-1.53)	0.029
	No	GG	24/86	-		1.00		16/86	-		1.00	
		GA	74/174	43.1	0.058	1.31(1.12-2.11)	0.024	61/174	52.5	0.031	1.59(0.91-2.79)	0.103
		AA	38/90	39.0	0.022	1.36(1.14-2.29)	0.026	33/90	34.5	0.011	1.86(1.01-3.43)	0.047
		GA+AA	112/264	39.0	0.034	1.33(1.15-2.09)	0.022	94/264	44.5	0.015	1.67(1.27-2.87)	0.023
Histology	SCC	GG	14/31	-		1.00		7/31	-		1.00	
		GA	55/87	22.7	0.422	1.28(0.71-2.30)	0.416	34/87	62.8	0.455	1.32(0.59-2.99)	0.501
		AA	23/40	23.5	0.637	1.15(0.59-2.42)	0.674	16/40	36.7	0.367	1.59(0.65-3.87)	0.308
		GA+AA	78/127	22.5	0.461	1.24(0.70-2.19)	0.464	50/127	52.9		1.40(0.63-3.09)	0.407
	ADC	GG	30/80	43.1		1.00		24/80	-		1.00	
		GA	111/186	20.7	0.008	1.72(1.15-2.58)	0.008	92/186	35.9	0.049	1.57(0.99-2.45)	0.051
		AA	50/92	22.5	0.051	1.57(1.00-2.47)	0.052	42/92	39.2	0.055	1.65(1.01-2.73)	0.050
		GA+AA	160/278		0.009	1.67(1.31-2.47)	0.010	134/278	36.4		1.59(1.03-2.46)	0.036
	Other	GG	14/37	30.8		1.00		12/37	52.9		1.00	
		GA	53/108	28.9	0.216	1.45(0.81-2.61)	0.216	40/108	44.5	0.595	1.20(0.63-2.28)	0.585
		AA	33/59	23.4	0.025	1.99(1.06-3.72)	0.032	26/59	35.5	0.070	1.82(0.91-3.60)	0.088
		GA+AA	86/167	23.8	0.091	1.62(0.92-2.85)	0.096	66/167	41.7		1.38(0.75-2.56)	0.303
Stage	IIIA	GG	27/84	-		1.00		21/68	-		1.00	
		GA	76/168	39.0	0.114	1.34(1.16-2.09)	0.036	66/168	44.5	0.303	1.25(1.06-2.06)	0.037
		AA	39/81	26.7	0.106	1.35(1.08-2.22)	0.039	32/81	32.6	0.012	1.38(1.29-2.26)	0.014
		GA+AA	115/249	39.0	0.085	1.34(1.18-2.05)	0.014	98/249	37.5	0.008	1.29(1.18-2.09)	0.028
	IIIB	GG	31/64	28.9		1.00		22/64	47.9		1.00	
		GA	143/213	22.0	0.026	1.52(1.03-2.25)	0.034	100/213	55.6	0.127	1.44(0.91-2.29)	0.122
		AA	67/110	19.7	0.044	1.51(1.01-2.33)	0.043	52/110	24.9	0.038	1.68(1.02-2.78)	0.043
		GA+AA	210/323	21.8	0.020	1.52(1.04-2.22)	0.030	152/323	36.9	0.005	1.51(1.17-2.37)	0.021
Treatment	RT	GG	31/65	28.9		1.00		22/65	47.9		1.00	
		GA	139/208	21.5	0.033	1.50(1.02-2.22)	0.041	100/208	37.8	0.130	1.49(0.93-2.39)	0.096
		AA	64/107	19.7	0.066	1.45(1.39-2.23)	0.034	49/107	30.6	0.049	1.74(1.05-2.92)	0.034
		GA+AA	230/315	21.2	0.029	1.48(1.02-2.17)	0.041	149/315	35.9	0.021	1.57(1.21-2.48)	0.033
	CRT	GG	27/83	-		1.00		21/83	-		1.00	
		GA	80/173	29.6	0.100	1.31(0.80-2.16)	0.288	66/173	52.6	0.316	1.31(0.79-2.16)	0.288
		AA	42/84	26.7	0.036	1.51(1.27-2.62)	0.042	35/84	33.1	0.003	1.51(1.37-2.62)	0.014
		GA+AA	122/257	29.6	0.025	1.37(1.30-2.22)	0.017	101/257	43.5	0.019	1.37(1.25-2.22)	0.017
Does	≥65Gy	GG	32/76	32.9		1.00		23/76	-		1.00	
		GA	141/227	23.1	0.178	1.20(0.77-1.89)	0.423	101/227	40.4	0.695	1.19(0.71-2.01)	0.511
		AA	70/116	17.0	0.015	1.08(1.04-1.81)	0.025	52/116	29.2	0.016	1.21(1.07-2.17)	0.023
		GA+AA	211/343	22.5	0.023	1.16(1.05-1.80)	0.038	153/343	35.6	0.028	1.19(1.02-1.98)	0.036
	<65Gy	GG	26/72	-		1.00		20/72	-		1.00	
		GA	78/154	27.1	0.009	1.62(1.09-2.42)	0.018	65/154	52.6	0.075	1.35(0.85-2.15)	0.203
		AA	36/75	39.0	0.003	1.69(1.09-2.61)	0.018	32/75	26.9	0.006	1.69(1.02-2.79)	0.042
		GA+AA	114/229	29.4	0.003	1.64(1.11-2.42)	0.012	97/229	33.7	0.024	1.45(1.39-2.28)	0.015

*P^a^*, Log-rank P; *P^b^*, multivariate cox regression; MST, median survival time; RT, radiation therapy; CRT, chemoradiation therapy.

**Table 5:** Subgroup multivariate analysis of clinical parameters for 189037 and survival outcomes.

**Parameters**	**Subgroup**	**Genotype**	**Disease-free survival**					**Overall survival**				
			**Event/No.**	**MST (month)**	***P^a^***	HR*(95%CI)	***P^b^***	**Event/No.**	**MST (month)**	***P^a^***	HR*(95%CI)	***P^b^***
rs189037												
Smoking	Yes	AA	24/71	-		1.00		24/71	43.5			
		AG	121/214	24.5	0.014	1.61(0.97-2.66)	0.063	122/214	34.6	0.055	1.31(0.78-2.19)	0.309
		GG	43/85	24.1	0.039	1.67(1.06-2.60)	0.023	37/85	31.8	0.166	1.90(1.22-2.95)	0.004
		AG+GG	164/299	24.5	0.012	1.65(1.08-2.55)	0.022	159/299	32.1	0.010	1.71(1.11-2.63)	0.015
	No	AA	26/94	59.3		1.00		21/94	-			
		AG	70/169	40.4	0.184	1.40(1.09-2.0)	0.046	70/169	36.9	0.095	1.08(0.58-2.03)	0.800
		GG	25/87	61.4	0.017	1.05(1.01-1.83)	0.035	19/87	-	0.027	1.78(1.09-2.90)	0.021
		AG+GG	95/256	44.5	0.015	1.29(1.13-2.00)	0.025	89/256	42.9	0.025	1.57(1.09-2.53)	0.045
Histology	SCC	AA	28/45	22.7		1.00		14/45	73.5		1.00	
		AG	49/80	23.5	0.836	0.62(0.33-1.16)	0.136	35/80	32.1	0.353	0.62(0.26-1.47)	0.276
		GG	15/33	-	0.153	0.95(0.60-1.51)	0.832	8/33	-	0.383	1.34(0.72-2.49)	0.354
		AG+GG	64/113	22.7	0.110	1.56(0.90-2.72)	0.115	43/113	38.1	0.067	1.98(0.64-4.190	0.073
	ADC	AA	51/90	22.5		1.00		33/90	64.3		1.00	
		AG	107/178	20.7	0.034	1.73(1.17-2.56)	0.006	102/178	31.5	0.089	0.65(0.38-1.11)	0.653
		GG	33/90	43.1	0.007	1.80(1.16-2.78)	0.009	23/90	58.4	0.000	1.46(0.99-2.170	0.057
		AG+GG	140/268	21.5	0.003	1.75(1.20-2.55)	0.003	125/268	34.6	0.002	2.01(1.29-3.13)	0.002
	Other	AA	15/37	33.3		1.00		9/37	-		1.00	
		AG	65/125	28.9	0.271	0.92(0.55-1.51)	0.729	55/125	36.9	0.318	1.41(0.61-3.25)	0.426
		GG	20/42	24.7	0.154	0.64(0.33-1.25)	0.194	14/42	-	0.995	1.80(0.89-3.65)	0.101
		AG+GG	85/167	30.8	0.505	0.85(0.52-1.38)	0.508	69/167	37.8	0.629	1.15(0.65-2.06)	0.631
Stage	IIIA	AA	25/90	58.6				22/90	-			
		AG	77/162	60.2	0.051	1.09(0.62-1.91)	0.760	77/162	46.0	0.085	1.07(0.58-1.97)	0.832
		GG	25/81	33.9	0.037	1.59(1.01-2.50)	0.044	20/81	-	0.012	1.85(1.15-2.97)	0.011
		AG+GG	102/243	39.0	0.012	1.43(1.31-2.22)	0.037	97/243	42.7	0.045	1.62(1.02-2.57)	0.042
	IIIB	AA	25/75	43.7		1.00		23/75	44.3		1.00	
		AG	114/221	30.1	0.032	1.59(1.02-2.48)	0.042	115/221	40.2	0.250	1.35(0.80-2.28)	0.267
		GG	43/91	27.1	0.037	1.45(1.28-2.39)	0.031	36/91	33.2	0.009	1.86(1.18-2.94)	0.008
		AG+GG	157/312	27.1	0.028	1.55(1.05-2.38)	0.029	151/312	33.7	0.019	1.69(1.09-2.65)	0.020
Treatment	RT	AA	23/71	-		1.00		22/71	48.4		1.00	
		AG	110/218	28.1	0.027	1.54(0.98-2.43)	0.059	111/218	41.7	0.167	1.39(0.82-2.36)	0.221
		GG	44/91	30.1	0.019	1.49(1.19-2.48)	0.021	38/91	34.9	0.026	1.78(1.12-2.82)	0.015
		AG+GG	154/309	29.0	0.035	1.53(1.08-2.38)	0.049	149/309	34.9	0.036	1.65(1.06-2.59)	0.028
	CRT	AA	27/94	61.4		1.00		23/94	-		1.00	
		AG	81/165	33.6	0.916	1.09(0.62-1.89)	0.772	81/165	35.1	0.869	0.99(0.53-1.85)	0.987
		GG	24/81	-	0.029	1.58(1.02-2.46)	0.040	18/81	-	0.003	1.87(1.17-2.97)	0.009
		AG+GG	105/246	34.5	0.039	1.43(1.02-2.20)	0.042	99/246	36.9	0.029	1.61(1.02-2.54)	0.041
Does	≥65Gy	AA	24/85	-		1.00		23/85	48.4		1.00	
		AG	113/232	29.0	0.069	1.17(0.80-1.73)	0.420	114/232	41.7	0.799	1.23(0.82-1.86)	0.311
		GG	46/102	30.1	0.014	1.31(1.23-1.32)	0.017	39/102	24.4	0.010	1.17(1.07-1.32)	0.020
		AG+GG	159/334	29.2	0.013	1.25(1.18-1.93)	0.021	153/334	34.9	0.006	1.47(1.29-2.36)	0.013
	<65Gy	AA	26/80	59.3		1.00		22/80	-		1.00	
		AG	78/151	61.4	0.016	1.68(1.02-2.76)	0.041	78/151	35.5	0.133	1.54(0.91-2.58)	0.104
		GG	22/70	33.6	0.039	1.70(1.09-2.66)	0..019	17/70	-	0.007	1.98(1.26-3.11)	0.003
		AG+GG	100/221	38.7	0.014	1.70(1.10-2.61)	0.017	95/221	36.9	0.013	1.84(1.18-2.86)	0.007

*P^a^*, Log-rank P; *P^b^*, multivariate cox regression; MST, median survival time; RT, radiation therapy; CRT, chemoradiation therapy.

## DISCUSSION

In this study, we assessed the association between four *ATM* SNPs and the survival outcomes in NSCLC patients of Han Chinese origin who received radiation therapy alone or chemo-radiation therapy. We demonstrated that two SNPs, rs664143 and rs189037, were significantly associated with DFS and OS rates in NSCLC patients treated with radiation or chemoradiation therapy. The association was significant with a prior false-positive rate of 0.1. NSCLC patients with rs664143 G or rs189037A alleles were at increased risk of disease progression compared with the other SNP genotypes. These results were further confirmed by the subgroup analysis. These findings suggest that these two *ATM* variants are associated with the prognosis of NSCLC patients and determine the efficacy of radiation therapy.

Radiation therapy induces DNA damage and tumor cell death. Inactivating *ATM* gene mutations contribute to genomic instability by reducing the efficiency of DNA double-strand break repair in response to radiation. *ATM* protein also detects DNA damage or other abnormal DNA structures in response to radiations and initiates DNA damage repair response that may include cell cycle arrest or apoptosis [21]. *ATM* activates checkpoint kinase, which induces phosphorylation of CDC25 at Ser216 and inhibits the cell cycle progression by suppressing the activity of CDC25 and M-Cdk. ATM also regulates the activity of p53, a critical tumor suppressor protein in multiple ways. It mediates p53 phosphorylation through Chk2 kinase activation. Moreover, ATM phosphorylates Mdm2 proto-oncogene and prevents its binding to p53. Furthermore, activation of p53 induces the expression of p21, which blocks the cell cycle in the G1-S phase by inhibiting CDK activity [22, 23]. Therefore, inactivating mutations in the *ATM* gene can disrupt these critical cellular mechanisms. *ATM* inhibition enhances the sensitivity of cancer cells to radiation therapy [24, 25], whereas, phosphorylated ATM protein increases the radiation resistance of cancer cells and correlates with poor prognosis of cancer patients [26]. Considering the importance of *ATM* kinase in DNA damage repair, our data suggests that activating or inactivating *ATM* gene polymorphisms influence the efficacy of radiation therapy and the prognosis of NSCLC patients.

Previous studies have shown that *ATM* gene polymorphisms are associated with poor prognosis of patients with pancreatic cancer, acute myeloid leukemia, and colorectal cancer [27, 28, 29]. Su et al. performed a case-control study of 230 NSCLC patients and showed that *ATM* rs664143 was not associated with the treatment response of patients with advanced NSCLC [30]. However, they did not evaluate its relationship with the prognosis of NSCLC patients. Du et al. showed that *ATM* rs664143 A and rs664677 C alleles were associated with poor prognosis of 412 esophageal squamous cell carcinoma (ESCC) patients that received radiation or chemoradiation therapy [31]. In the present study, we demonstrate that NSCLC patients with the rs664143 A allele is associated with unfavorable prognosis after receiving radiation or chemoradiation therapy. One probable mechanism explaining these outcomes is inaccurate splicing when rs664143 is combined with an intronic splicing enhancer or repressor [32]. However, further investigation is required to ascertain the molecular mechanisms involved in the process. We also demonstrate that *ATM* rs189037 is associated with poor prognosis of NSCLC patients treated with radiation or chemoradiation therapy. This SNP is located in the promoter region of the *ATM* gene. A previous study showed that *ATM* rs189037 is associated with radiation- induced pneumonia in lung cancer patients [33], but, its role in survival outcomes of NSCLC patients has not been reported. In a previous study of breast cancer patients, those with *ATM* rs664677 TC genotype showed increased radiation resistance compared to the rs664677 TT genotype [34]. Furthermore, pancreatic cancer patients with the rs664677 TC genotype showed worse prognosis than those with the rs664677 TT genotype [28]. In advanced ESCC patients receiving radiation therapy, two *ATM* SNPs, rs664143, and rs664677, were associated with survival times [31]. In contrast, our study showed no correlation between rs664677 and prognosis of NSCLC patients that received radiation or chemoradiation therapy. The reasons for these contrasting findings are not clear and further studies are necessary to determine the differential function or role of rs664677 in different types of cancers.

One plausible explanation for our findings is that the two ATM SNPs (or haplotypes) modulate the function of the ATM protein. This determines the response of NSCLC cells to radiation and chemo-radiation therapy, which affects their survival and contributes to differential prognosis of patients with different *ATM* SNPs. ATM protein is a key regulator of cell cycle. Cell cycle is blocked under conditions of DNA damage, incomplete replication, or abnormal spindle formation. When normal cells are irradiated, DNA repair mechanisms are activated so that the DNA damage is rectified. Cell cycle inhibition of irradiated cells prevents the proliferation of malignant cells that contain genetic mutations. However, tumor cells are defective in cell cycle checkpoints and DNA repair mechanisms. This results in uncontrolled proliferation and differentiation of tumor cells with genetic mutations [35]. Mutations in the *ATM* gene alter the structure and function of the ATM protein in ataxia telangiectasia (A-T) patients. Therefore, A-T patient cells show aberrant cell cycle checkpoints and DNA damage repair, increased sensitivity to apoptosis, chromosomal instability, and radiation sensitivity. Moreover, the incidence of cancers is significantly higher in patients with heterozygous or homozygous *ATM* mutations compared to individuals with the wild-type *ATM* gene [36]. The rs189037 is located at the 5'UTR of ATM gene (NC_000011.10: g. 108354934), and rs664143 is located at the intron of ATM gene (NC_000011.10: g.108354934). Two SNPs are very close loci (131bp) existing in two intros. We used the web-based tool (Improbizer, https://users.soe.ucsc.edu/~kent/improbizer/improbizer.html) to check the splicing effect of two sites, and the results suggested that two sited exist in protein-binding motifs have a potential as binding sites of intronic splicing enhancer, indicating a possibility that both sites may be related to splicing process to lead to inaccurate splicing. However, this hypothesis should be confirmed through further research.

Our study has several limitations. Firstly, our study includes NSCLC patients treated with radiation or chemoradiation, but does not investigate NSCLC patients that underwent surgery. Secondly, we adjusted the survival outcomes for a few common clinical parameters, but, more factors should be considered. Thirdly, we did not identify the mechanisms underlying the findings of our study. Finally, we did not perform haplotype analysis because only two significant SNPs were found.

In conclusion, our study demonstrates that *ATM* gene polymorphisms are significantly associated with disease progression and survival outcomes in NSCLC patients that have received radiation or chemoradiation therapy. These two *ATM* SNPs are potential prognostic biomarkers to predict survival outcomes of NSCLC patients that receive radiation or chemoradiation therapy.

## MATERIALS AND METHODS

### Study population

This two-center follow-up study was conducted at Yantai Yuhuangding Hospital, the Affiliated Hospital of Qingdao University and Tangshan People’s Hospital from January 2009 to December 2017. The inclusion criteria were: (1) newly diagnosed Han Chinese NSCLC patients that were confirmed by biopsy with TNM stage IIIA–IIIB NCSLC tumors; (2) availability of follow-up clinical data, and (3) patients underwent radiotherapy or chemoradiotherapy. Patients that (1) underwent surgery or stereotactic ablative radiation therapy, and (2) with an history of lung cancer and recurrent disease, severe cardiovascular diseases, cerebral apoplexy, or depression were excluded from the study. Tumors were classified based on histology according to the World Health Organization system for NSCLC. The tumor staging was according to the revised American Joint Committee on Cancer/Union for International Cancer Control (AJCC/UICC 7^th^ Edition). This study was approved by the Ethics Committee of Yantai Yuhuangding Hospital, the Affiliated Hospital of Qingdao University, and conducted in accordance with the World Medical Association Declaration of Helsinki. We obtained written informed consent from all study subjects.

### Radiation therapy

All patients underwent computed tomography (CT) scans of 5 mm slice thickness in a supine position with hands crossed before placing the forehead and thermoplastic body model fixation. The scans were performed from the mastoid process to the bottom of the lung and the images were transmitted over the network to a three-dimensional (3-D) treatment planning system. The target area delineation was according to ICRU50 and ICRU62 report guidelines. The gross tumor volume (GTV) was defined as the tumor volume seen below the lung window. The GTV node (GTVnd) was defined as a metastatic lymph node seen below the mediastinal window. The characteristics of the GTVnd were: short diameter > LCM; multiple fusions; necrosis or envelope invasion; and confirmed by positron emission tomography (PET) or mediastinoscopy. For concurrent chemoradiotherapy or radiotherapy alone, the double-lung V20 was limited to < 28% and < 30%, respectively, and adjusted according to the patient’s physical condition, age, complications, and basic lung-function parameters. The other tissue limits included a maximum dose of the spinal cord of < 45 Gy, a cardiac V40 of < 30%, and an esophageal V50 of < 40–50%. A physician confirmed the treatment plan developed by the radiologist. The plan was further verified by calibration on the CT simulation positioning machine. Finally, the treatment was performed using a Vanaii linear accelerator.

### Clinical data and follow-up

We obtained clinical data from the medical records of the patients for parameters such as age at treatment, gender, smoking, histologic type, TNM stage, treatment method, radiation technique, and radiation dose. The survival data was collected by follow-up through the telephone or from the outpatient medical records. The patients underwent physical examinations, CT or PET/CT, laboratory tests, and lung function evaluations at 6 weeks after therapy. The follow-up interval was every 3 months for 2 years, and 6 months thereafter. The primary follow-up outcome was overall survival (OS). OS was defined as the time period from the start of treatment to the last follow-up or death. We also evaluated disease-free survival (DFS), which was defined as the time period from the start of treatment until the date of the first local recurrence or metastasis at the last follow-up. Patients without progression were censored at the last follow-up date.

### Selection and genotyping of *ATM* SNPs

We first searched for the *ATM* gene SNPs in the dbSNP database (https://www.ncbi.nlm.nih.gov/snp/) using a minor allele frequency of > 5%. Next, we selected tagSNPs with r^2^ > 0.8 among the Han Chinese population using the International HapMap project database and identified four SNPs (rs664143, rs664677, rs189037, and rs373759) with moderate linkage disequilibrium by analyzing the 1000GENOMES project database.

We obtained 5 mL of fasting venous blood in ethylenediaminetetraacetic acid (EDTA)-coated tubes and isolated the genomic DNA from peripheral blood leukocytes using the Biospin Whole Blood Genomic DNA Extraction Kit (Bioer Technology Co., Ltd., China) according to the manufacturer’s instructions. The samples were stored at −20°C.

The SNP sequences were PCR amplified using primer sequences that were designed using Primer Premier 5.0, and synthesized by Sangon Biotech (Shanghai, China) as shown in Supplementary Table 1. The PCR protocol was: initial denaturation cycle at 95°C for 7 min; 35 cycles of denaturation at 95°C for 1 min, annealing at 56°C for 1 min, and extension at 72°C for 1 min; final extension cycle at 72°C for 10 min. The PCR products were separated by 2% agarose gel electrophoresis, purified by ExoSAP-IT (USB Corp., Cleveland, OH, USA), sequenced in an Applied Biosystems 3730xl automated sequencer (Applied Biosystems, Foster City, CA, USA), and analyzed using the Vector NTI software.

### Statistical analysis

The continuous variables were converted into categorical variables based on the mean age or median dose. The median OS and DFS were determined by Kaplan-Meir survival curve analyses using the log-rank test. The relationship between clinical parameters, *ATM* SNPs and survival parameters, OS and DFS, was determined using univariate and multivariate cox proportional hazards regression models. The multivariate Cox proportional regression model was adjusted for age, gender, smoking, histology, stage, treatment, radiation technique, and dose and the corresponding hazard ratios (HRs) and 95% confidence intervals (CIs) were estimated. Subgroup analyses were performed for significant variables such as smoking, tumor stage, treatment, and radiation dose. The false-positive probability analysis was conducted by assuming that the HR for a risk allele was 1.5 and the HR for a protective allele was 1/1.5 times below the prior probability of 0.01 for each SNP. A significant association was defined as a false-positive value of < 0.20. Statistical significance was defined as a two-sided P-value < 0.05. Statistical analysis was performed using the SPSS 23.0 software.

## Supplementary Material

Supplementary Figure 1

Supplementary Table 1
