# Supplementary figures and images for: ATM gene polymorphisms are associated with poor prognosis of non-small cell lung cancer receiving radiation therapy
Source: Aging (Albany NY). 2020 Apr 24;12(8):7465–79. doi: 10.18632/aging.103094 (PMC7202543; doi:10.18632/aging.103094)

SUPPLEMENTARY FIGURE

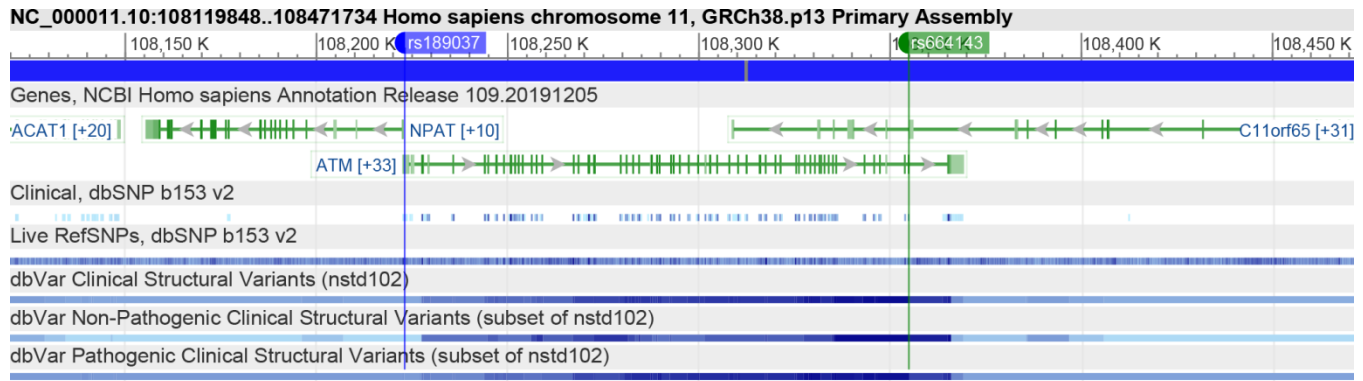

Supplementary Figure 1. Clinical structural variants of rs664143 and rs189037.

Supplement: Supplementary Figure 1 [file aging-12-103094-s002..pdf]
