# Supplementary material for: ATM gene polymorphisms are associated with poor prognosis of non-small cell lung cancer receiving radiation therapy
Source: Aging (Albany NY). 2020 Apr 24;12(8):7465–79. doi: 10.18632/aging.103094 (PMC7202543; doi:10.18632/aging.103094)
Supplement: Supplementary Table 1 [file aging-12-103094-s001..pdf]

SUPPLEMENTARY TABLE

Supplementary Table 1. PCR-RFLP primer sequences of four SNPs.

SNP	Primer sequence	Temp (°C)
rs664677	5'- GAAAGACATATTGGAAGTAACGTA	54
	5′- TTTCCTCTCCTTTGTTAGATGCCTG	
rs664143	5'- GAAAACTAGAGACTACTTACAATGC	55
	5'-TTCTTACCAGGTAGACTGTGTATCAC	
rs789037	5'- GCTGCTTGGCGTTGCTTC	60
	5'- CATGAGATTGGCGGTCTGG	
rs373759	5'-GGCCAGTTACCATCCCTCCAAT	59
	5'GCTGAGAGAGAAGGAAAAGTGAGGC	
